# Supplementary material for: Use of AbobotulinumtoxinA for Cosmetic Treatments in the Neck, and Middle and Lower Areas of the Face: A Systematic Review
Source: Toxins (Basel). 2021 Feb 22;13(2):169. doi: 10.3390/toxins13020169 (PMC7926328; doi:10.3390/toxins13020169)
Supplement: Supplementary file 1 [file toxins-13-00169-s001.pdf]

Supplementary Materials: Use of AbobotulinumtoxinA for Cosmetic Treatments in the Neck, and Middle and Lower Areas of the Face: A Systematic Review

Hassan Galadari, Ibrahim Galadari, Riekie Smit, Inna Prygova and Alessio Redaelli

Table S1. Summary of patient satisfaction and quality of life outcomes.

Study	Study Design	Country	Age, Years	Participants, *N* (*n* [%] Women, *n* [%] Men)	Area(s) of Injection. Indication Assessed	Assessment Tool Used	Key Findings
Awaida et al. 2018 [1]	Non-randomized controlled trial	Lebanon	Mean (SD): 55.9 (5.8)	25 (25 [100%] women)	Neck, lower face. Oral commissures, marionette lines, jowls, neck volume, platysmal bands at rest and at maximal contraction	Satisfaction survey using a 4-point scale (1, very satisfied; 2, satisfied; 3, dissatisfied; 4 very dissatisfied)	100% (*n* = 25) of patients were satisfied with the results after both the micro injections and Nefertiti techniques<br>88% (*n* = 22) of patients were willing to repeat the micro injections technique and recommend to a friend of family member<br>100% (*n* = 25) of patients were willing to repeat the Nefertiti technique and recommend to a friend of family member<br>When asked about their preferred technique for neck rejuvenation, 72% (*n* = 18) of patients chose the micro injections technique, 20% (*n* = 5) preferred the Nefertiti lift and 8% (*n* = 2) had no preference

Study	Study Design	Country	Age, Years	Participants, *N* (*n* [%] Women, *n* [%] Men)	Area(s) of Injection. Indication Assessed	Assessment Tool Used	Key Findings
Chang et al. 2018 [2]	Non-randomized controlled trial	USA	Mean: 51.7<br>Range: 28.8–72.4	32 (32 [100%] women)	Lower face.<br>Perioral rhytids, marionette lines, chin, nasolabial fold, oral commissures, cheeks	FACE-Q survey, including questions on satisfaction with facial appearance overall	On average, patients were 22.2% ($p = 0.014$) more satisfied with their overall facial appearance at day 14 compared with baseline
Hexsel et al. 2013 [3]	RCT	Brazil	Mean (SD): 48.3 (7.2)<br>Range: 30.0–60.0	85 (82 [96.5%] women; 3 [3.5%] men)	Mid, lower face.<br>Lower eyelid wrinkles, nasal wrinkles, malar wrinkles, perioral wrinkles, marionette lines, gummy/asymmetric smile, cellulitic chin	WHOQOL-BREF<br>Satisfaction and self-assessment questionnaire (SSQ) survey, to assess their level of satisfaction regarding the treatment: totally satisfied, satisfied, slightly satisfied, slightly dissatisfied, dissatisfied, totally dissatisfied	*QoL*<br>Scores on the physical domain of the WHOQOL-BREF questionnaire significantly improved between baseline and 4 weeks ($p = 0.036$)<br>Group 2 (who had a total dose of 166–205 U) had significantly higher mean scores for the physical and social relationships domains compared with group 1 (dose 120–165 U) ($p = 0.033$ and $p = 0.012$, respectively) and group 3 (dose 206–250 U) ($p = 0.004$ and $p = 0.025$, respectively). Scores on the psychological domain were also higher in group 2 than in group 3 (*p*-value not reported)<br><br>*Patient satisfaction*<br>96.4% of all participants were satisfied or totally satisfied at 4 weeks after treatment, 87% at 16 weeks

Study	Study Design	Country	Age, Years	Participants, *N* (*n* [%] Women, *n* [%] Men)	Area(s) of Injection. Indication Assessed	Assessment Tool Used	Key Findings
							after treatment, 84.7% at 20 weeks after treatment, and 95.3% at 24 weeks after treatment
Jabbour et al. 2017 [4]	Non-randomized controlled trial	Lebanon	Mean (SD): 54.8 (5.3)	30 (30 [100%] women)	Lower face, neck. Jowls, platysmal bands at rest and at maximal contraction, marionette lines, neck volume, oral commissures	Satisfaction survey using a 4-point scale (1, very satisfied; 2, satisfied; 3, dissatisfied; 4 very dissatisfied)	96.6% of participants were satisfied with results<br>93.3% rated themselves as improved (Subject Global Aesthetic Improvement scale)<br>90% would repeat the procedure and 93.3% would recommend to friend or family member
Kim et al. 2005 [5]	Non-randomized controlled trial	South Korea	Age ranges:<br>13–19 years (*n* = 10)<br>20–29 years (*n* = 293)<br>30–39 years (*n* = 70)<br>40–49 years (*n* = 9)	383 (355 [92.7%] women; 28 [7.3%] men)	Lower face. Masseter muscle	Satisfaction survey (details not reported in manuscript)	268 participants (70%) were greatly satisfied with the result and 88 (23%) were generally satisfied<br>356 participants (93%) felt positive about the outcome
Mazzuco and Hexsel 2010 [6]	Observational study	Brazil	NR	16 (NR)	Lower face. Gummy smile	Satisfaction survey (details not reported in manuscript)	14 of 16 participants (87.5%) were reasonably satisfied with the results

Abbreviations: NR, not reported; RCT, randomized controlled trial; SD, standard deviation; WHOQOL-BREF, World Health Organization Quality of Life-Brief Version questionnaire.

Table S2. Searches carried out in: Ovid MEDLINE, all segments, 1946 to present; Embase 1974 to 18 July 2019; and Cochrane (CDSR, DARE, CENTRAL, NHS EED, The HTA database, and ACP Journal Club) 23 August 2019.

No.	Searches	Type
1	(dysport or abobotulinumtoxin or abobotulinumtoxinA or abobotulinum or AboBoNT-A or Azzalure).mp.	AbobotulinumtoxinA terms
2	plastic surgery/ or esthetics/ or esthetic surgery/ or dental care/ or procedures/	Surgery terms
3	(face or facial or aesthetic or cosmetic or line* or wrinkle* or aging or code bar or nasolabial folds or drooping nasal or nose or decollete or decolletage or drooping mouth corner or depressor anguli oris or marionette or dimpled chin or chin or nasal root or bunny lines or gummy smile or gingival smile or platysma* or neck or nefertiti or masseter hypertrophy or bruxism or sternocleidomastoid).mp.	
4	1 and (2 or 3)	
5	Clinical trial/	Randomized controlled trials
6	Randomized controlled trial/	
7	Randomization/	
8	Single blind procedure/	
9	Double blind procedure/	
10	Crossover procedure/	
11	Placebo/	
12	Randomized controlled trial.tw.	
13	Rct.tw.	
14	Random allocation.tw.	
15	Randomly allocated.tw.	
16	Allocated randomly.tw.	
17	(allocated adj2 random).tw.	
18	Single blind$.tw.	
19	Double blind$.tw.	
20	((treble or triple) adj blind$).tw.	
21	Placebo$.tw.	
22	Prospective study/	
23	or/5-22	
24	(real world or RWE).mp.	Observational studies
25	(cohort or observational).mp.	
26	exp Longitudinal Studies/ or longitudinal.mp.	
27	exp Retrospective Studies/	
28	or/24-27	Exclusions and Boolean combinations
29	Case study/	
30	Case report.tw.	
31	Abstract report/ or letter/	
32	or/29-31	
33	4 and (23 or 28)	
34	33 not 32	
35	limit 34 to (books or chapter or editorial or letter or note or "review" or short survey or tombstone)	
36	34 not 35	

*Anatomical units of the lower third.

Table S3. Eligibility criteria used in the systematic literature review.

	Inclusion Criteria	Exclusion Criteria
Population	Patients receiving abobotulinumtoxinA injections in the neck or middle or lower areas of the face for aesthetic purposes	Patients receiving abobotulinumtoxinA injections for other indications or in other regions of the face and body
Interventions	AbobotulinumtoxinA	OnabotulinumtoxinA<br>IncobotulinumtoxinA
Comparator	Placebo<br>Other neurotoxins<br>Cosmetic treatments	
Outcomes[a]	Treatment approaches (treatment guidelines, injection practice and technique, injection frequency)<br>Efficacy outcomes (onset and duration of action)<br>Safety outcomes (adverse events)<br>Patient outcomes (cosmetic muscle relaxant, wrinkle or line assessments, patient satisfaction, quality of life)	No relevant outcomes
Study Design	Observational studies (retrospective, cross-sectional or prospective studies)[b]<br>RCTs<br>Non-randomized controlled trial[c]	Case studies or series<br>Modeling study<br>Comment/editorial<br>Narrative reviews
Countries	Not restricted by country or region	
Date Restriction	Not restricted by date	

Abbreviations: RCT, randomized controlled trial. [a] Excludes outcomes for treatment of non-cosmetic conditions such as hemifacial spasm, chronic facial pain, rhinitis, facial dystonia, sialorrhea, synkinesis, masseteric hypertrophy. [b]Relevant observational studies, non-randomized controlled trials and RCTs, which were referenced in reviews identified in the searches, were included. [c] Narrative reviews were included at first screening so that relevant studies referenced in the reviews could be identified. Narrative reviews were excluded at full-text review.

References

1. Awaida, C.J.; Jabbour, S.F.; Rayess, Y.A.; El Khoury, J.S.; Kechichian, E.G.; Nasr, M.W. Evaluation of the microbotox technique: an algorithmic approach for lower face and neck rejuvenation and a crossover clinical trial. *Plastic & Reconstructive Surgery* **2018**, *142*, 640–649, doi:https://dx.doi.org/10.1097/PRS.0000000000004695.
2. Chang, C.S.; Chang, B.L.; Lanni, M.; Wilson, A.J.; Beer, J.; Percec, I. Perioral rejuvenation: a prospective, quantitative dynamic three-dimensional analysis of a dual modality treatment. *Aesthet* **2018**, *38*, 1225–1236, doi:https://dx.doi.org/10.1093/asj/sjy060.
3. Hexsel, D.; Brum, C.; Porto, M.D.; Soirefmann, M.; Siega, C.; Schilling-Souza, J.; Rodrigues, T.C. Quality of life and satisfaction of patients after full-face injections of abobotulinum toxin type A: a randomized, phase IV clinical trial. *Journal of Drugs in Dermatology: JDD* **2013**, *12*, 1363–1367.
4. Jabbour, S.F.; Kechichian, E.G.; Awaida, C.J.; Tomb, R.R.; Nasr, M.W. Botulinum toxin for neck rejuvenation: assessing efficacy and redefining patient selection. *Plastic & Reconstructive Surgery* **2017**, *140*, 9e–17e, doi:https://dx.doi.org/10.1097/PRS.0000000000003429.
5. Kim, N.H.; Chung, J.H.; Park, R.-H.; Park, J.B. The use of botulinum toxin type A in aesthetic mandibular contouring. *Plastic & Reconstructive Surgery* **2005**, *115*, 919–930, doi:10.1097/01.PRS.0000153236.79775.A0.
6. Mazzuco, R.; Hexsel, D. Gummy smile and botulinum toxin: a new approach based on the gingival exposure area. *J Am Acad Dermatol* **2010**, *63*, 1042–1051, doi:https://dx.doi.org/10.1016/j.jaad.2010.02.053.
